# Technological Advances in Pre-Operative Planning

**DOI:** 10.3390/jcm14155385

**Published:** 2025-07-30

**Authors:** Mikolaj R. Kowal, Mohammed Ibrahim, André L. Mihaljević, Philipp Kron, Peter Lodge

**Affiliations:** 1 Leeds Institute of Medical Research, University of Leeds, Leeds LS9 7TF, UK; 2 University Clinic for General, Visceral and Transplantation Surgery, University Hospital Tübingen, 72076 Tübingen, Germany

**Keywords:** robotic surgery, artificial intelligence in surgery, surgical training, AR in surgery, 3D imaging, surgical navigation, extended realities

## Abstract

Surgery remains a healthcare intervention with significant risks for patients. Novel technologies can now enhance the peri-operative workflow, with artificial intelligence (AI) and extended reality (XR) to assist with pre-operative planning. This review focuses on innovation in AI, XR and imaging for hepato-biliary surgery planning. The clinical challenges in hepato-biliary surgery arise from heterogeneity of clinical presentations, the need for multiple imaging modalities and highly variable local anatomy. AI-based models have been developed for risk prediction and multi-disciplinary tumor (MDT) board meetings. The future could involve an on-demand and highly accurate AI-powered decision tool for hepato-biliary surgery, assisting the surgeon to make the most informed decision on the treatment plan, conferring the best possible outcome for individual patients. Advances in AI can also be used to automate image interpretation and 3D modelling, enabling fast and accurate 3D reconstructions of patient anatomy. Surgical navigation systems utilizing XR are already in development, showing an early signal towards improved patient outcomes when used for hepato-biliary surgery. Live visualization of hepato-biliary anatomy in the operating theatre is likely to improve operative safety and performance. The technological advances in AI and XR provide new applications in pre-operative planning with potential for patient benefit. Their use in surgical simulation could accelerate learning curves for surgeons in training. Future research must focus on standardization of AI and XR study reporting, robust databases that are ethically and data protection-compliant, and development of inter-disciplinary tools for various healthcare applications and systems.

## 1. Introduction

Despite centuries of advances in medical knowledge, technology and peri-operative care, surgery remains a healthcare intervention with substantial risks for patients. Global data shows that 16.8% of patients undergoing surgery will develop at least one surgical complication, with post-operative mortality accounting for 7.7% of worldwide deaths [1,2]. For complex surgery, such as hepato-biliary surgery, the risks are even higher. Its nature of variable anatomy coupled with locally invasive malignancies increases the risk of surgical complications, reported as high as 48% and 60% for pancreatic and liver resections, respectively [3,4]. There is an urgent need to improve peri-operative care to reduce the global burden of surgical morbidity and mortality. Central to peri-operative care is clinical decision-making, often taking place at diagnosis, surgical planning meetings or in the operating theatre. Novel technologies can now provide clinicians with more up to date and detailed information to enhance patient selection, surgical planning and safety in the operating theatre.

The era of digital surgery, also known as surgery 4.0, has introduced new technologies available to the surgeon in the operative workflow [5]. The rapid adoption of robotic surgery, artificial intelligence (AI) solutions and extended reality (XR) technology provides new tools for clinical practice [6]. These offer an opportunity to enhance every aspect of pre-operative planning, from image interpretation at diagnosis to surgical navigation systems in the operating theatre, improving safety and clinical outcomes for patients. In this review, we focus on the technological advances in pre-operative planning for hepato-biliary surgery. The aims are to display the latest solutions to enhance pre-operative planning and to discuss their route to routine practice adoption.

## 2. Foundations of Pre-Operative Planning

The risks associated with hepato-biliary surgery have always necessitated ample surgical planning in the peri-operative pathway. Appropriate patient selection is key to ensuring safety for patients. In surgery for primary and secondary liver malignancies, post-hepatectomy liver failure (PHLF) occurs in 9–30% of extended resections and it is a major determinant of post-operative death [7]. Accordingly, international guidelines provide a clinical algorithm to assist in identifying patients at risk of PHLF (evidence derived from high quality cohort studies) [8]. This includes functional assessments, which may involve computational mathematical scores that combine clinical parameters, and volumetry, assessing liver volume quantitatively through image analysis. These established approaches to pre-operative planning are limited by static models, which limit their potential accuracy. They are also based on a small number of clinical parameters, potentially missing essential information, such as nutritional status or sarcopenia on an individual patient level. The lack of comparison between the accuracy of functional assessments has been recognized as an area requiring further research. The current methods of volumetry also have their limitations. These frequently involve manual methods of image segmentation, a process which entails manually partitioning an image into distinct regions, in this case selecting the future liver remnant (FLR) for volumetric calculations [9]. This introduces inter-observer variability and relies heavily on radiological expertise and resources to deliver accurate risk predictions of PHLF. There is a need to standardize and automate risk prediction for patients undergoing hepato-biliary surgery.

Another key aspect of pre-operative planning in hepato-biliary surgery is imaging. Multiple modalities are used to plan operations. In the example of liver resections, magnetic resonance imaging (MRI) or computed tomography (CT) are used to detect deep liver lesions. On the other hand, ultrasound (US) is more accurate in detecting peripheral lesions close to the liver capsule, showing the complexity in imaging use for hepato-biliary surgery. The importance of appropriate imaging pathways was recently demonstrated in the CAMINO trial [10]. The randomized controlled trial investigated the addition of liver contrast-enhanced MRI for detection of colorectal liver metastasis (CRLM), showing a change in the local treatment plan in 31% (*n* = 92/298) of patients due to the MRI results. Standardized imaging protocols with appropriate modalities can therefore enhance pre-operative workflows. The amount of meaningful information obtained from imaging could be improved with image fusion, combining multiple modalities that capture all required detail for pre-operative planning.

**Figure 1:**
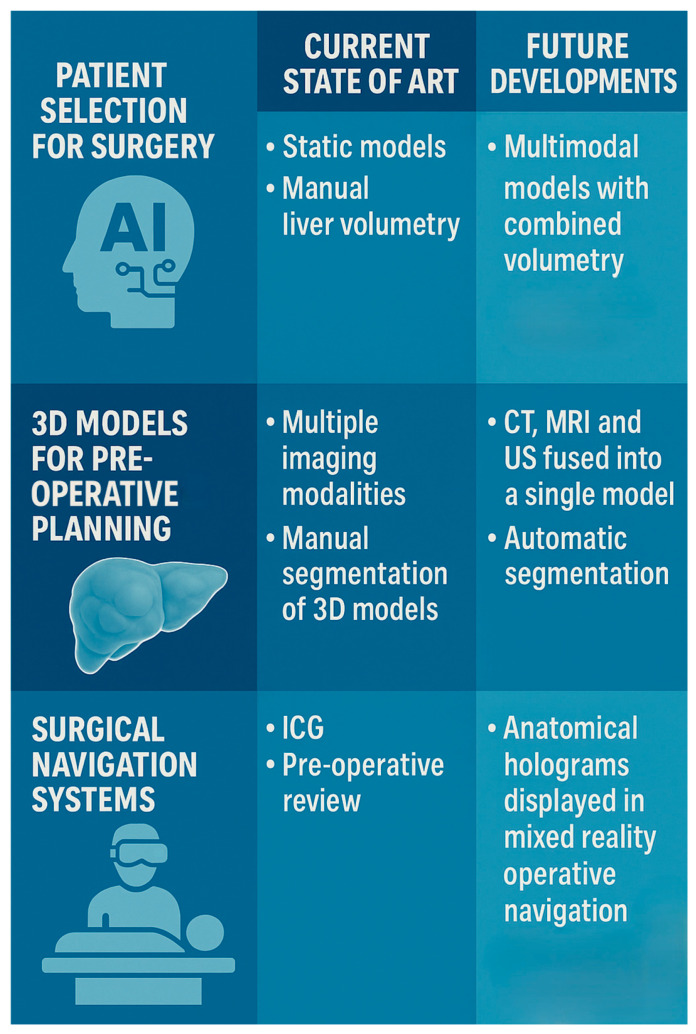
Innovation in pre-operative planning.

The role of pre-operative planning in hepato-biliary surgery is also important to anticipate challenging anatomy. Biliary anatomy is well known for its variability, with studies showing variant rates of over 40% between individuals [11]. A feared complication associated with this involves bile duct injury (BDI), occurring in approximately 3 in 1000 cholecystectomies [12]. Despite the introduction of the “critical view of safety”, adoption of new laparoscopic and robotic techniques has led to an increase in BDI incidence [13,14]. Novel imaging methods have enabled real-time visualization of biliary anatomy, such as indocyanine green (ICG) administration. When used during laparoscopic cholecystectomy, ICG has been shown to reduce operative time, conversion to open surgery rates, length of stay and BDI rates [15]. Although ICG now provides a mode of real-time visualization, it is limited by poor performance in certain conditions (such as presence of impacted gallstones or cystic duct stones) and its need for administration, which is most optimal the day before surgery [16]. The ideal solution for hepato-biliary surgery involves a method of biliary visualization that is on-demand, real-time and fused with existing surgical platforms. In summary, the current methods for pre-operative planning involve static models for risk prediction and a variety of imaging modalities, requiring clinicians to interpret and extract data from images manually. The use of pre-operative imaging for intra-operative navigation remains underutilized. The technologies with the most potential to enhance pre-operative planning in hepato-biliary surgery include AI, 3D visualization and XR, and are discussed as part of this review (Figure 1).

## 3. Generative Artificial Intelligence for Pre-Operative Planning

Generative artificial intelligence (GenAI) is set to revolutionize many industries and is rapidly gaining applications in healthcare [17]. This technology is characterized by extraction, structuring, analysis and synthesis of large datasets. GenAI is a specific domain within deep learning which is able to generate various outputs, such as text, images or code, outside of predefined constraints [18]. A recent development within GenAI set to increase its use is natural language processing, enabling computers to understand and manipulate text and speech [19]. This has led to the advent of GenAI chatbots, which in surgery are already being used to analyze data, automate literature reviews and draft surgical academic manuscripts [20]. These developments allow the surgeon to interact with GenAI which is now capable to learn from various forms of data. One of the newest forms of a chatbot was trained on available text from the internet, using a total of 45 terabytes of data [21]. There is an unprecedented opportunity to harness the power of GenAI to process large clinical datasets and generate outputs that can enhance pre-operative planning.

### 3.1. Predictive Analytics for Risk Stratification

The current difficulties in patient selection for hepato-biliary surgery lie with the heterogeneity of liver disease. PHLF risk is associated with a multitude of clinical variables including a rise in nonalcoholic steatohepatitis, patient factors that change dynamically and acquired dysfunction such as chemotherapy-associated liver injury (CALI) [22]. Individual models have been developed to assist in risk stratification, such as the APRI + ALBI model which can predict clinically significant PHFL with an AUC of 0.77 [23]. This captures the biochemical hepatic function, without a metric expression of individual liver structure and health. The accuracy could be improved using integrative models that combine quantitative volumetry with hepatic function models. A signal towards this has already been shown in early studies. Mai et al. developed an artificial neural network model (a type of machine learning involved in GenAI) to predict severe PHLF for patients undergoing a hemihepatectomy for hepatocellular carcinoma (HCC) [24]. Using clinical variables and combined volumetry, the model was able to predict severe PHLF with an AUC of 0.880, in their retrospective cohort study. Similar models are also being developed to predict outcomes in patients with pancreatic cancer [25]. This shows the potential for GenAI to improve risk prediction. Further variables could be integrated to increase the accuracy, such as live patient data on physical fitness from wearable devices or up-to-date liver imaging. Clinical teams will be empowered with GenAI-powered predictive analytics to make the best decisions for patients.

In addition to single outputs that inform decisions, GenAI could assist with complex decision-making in hepato-biliary surgery. GenAI technology is the perfect solution to process different data forms into a clinician-facing output that can be used in risk prediction and patient selection for hepato-biliary surgery. The solution could be used routinely in multi-disciplinary tumor (MDT) board meetings. The chatbot ChatGPT 4.0 (OpenAI, San Francisco, CA, USA) was recently tested as part of a MDT meeting for colorectal cancer [26]. The GenAI system was able to deliver appropriate treatment plans when provided with 157 real-world cases. The highest degree of concordance was seen for post-operative decisions (kappa coefficient = 0.876, *p* < 0.001), with lowest agreement in pre-operative decisions (kappa coefficient = 0.271, *p* = 0.003). The authors explained this difference by the lack of consensus between institutions on neoadjuvant and surgery-first pathways. It is likely that hepato-biliary decisions, particularly on neoadjuvant and surgery-first approaches to liver tumors, will face similar problems. However, the capacity for GenAI to process more data should overcome the initial disagreements that may occur. The result could involve efficient, on-demand and accurate decision-making for patients with a new diagnosis of hepato-biliary malignancy, fast-tracking to the most optimal treatment to improve their outcomes. The GenAI solution would work 24 h per day, appropriately selecting treatment options for patients at the time of diagnosis. The potential to improve outcomes in hepato-biliary malignancy through this is substantial.

### 3.2. AI-Driven Imaging

**Figure 2:**
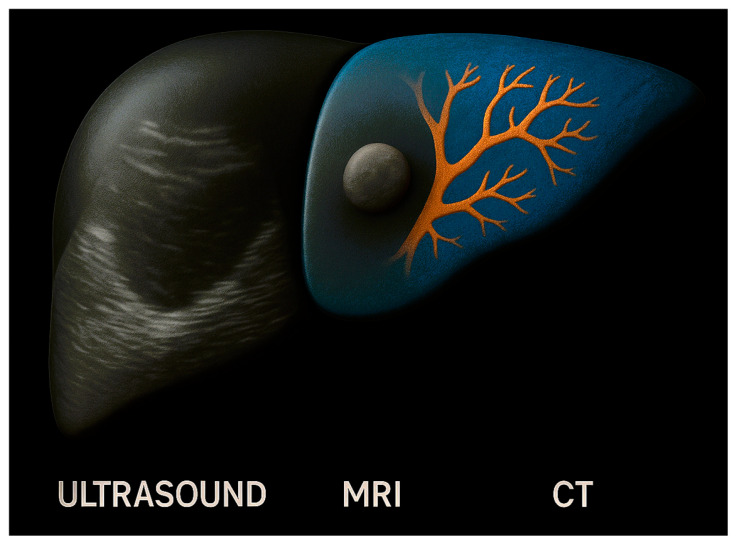
Illustration of imaging fusion for hepatic anatomy.

Imaging is key to planning hepato-biliary surgery, and each modality has its own strengths and limitations. The combination of CT, MRI and US into one 3D image would enable clinicians to visualize hepato-biliary anatomy in the best possible detail (Figure 2). Although this concept has been present for years, limitations in image processing have prevented the wider adoption of image fusion in clinical practice [27]. For hepato-biliary anatomy, discrepancies in viscera motility, respiration, heart rate and body movement between images can make co-registration of different modalities challenging. Manually overlaying images, such as MRI images for bile ducts against vascular structures from a CT scan, can be a time- and resource-consuming process. Advances in AI now provide learning-based methods which can automatically perform image fusion, improving the accuracy, adaptability and speed of co-registration for different modalities [28]. The learning-based methods can account for discrepancies between images to create one fused 3D reconstruction. Soon, clinicians could create 3D reconstructions of hepato-biliary anatomy using multiple imaging modalities at the click of a button. It is possible that the use of fused imaging will generate positive changes to patient pathways and outcomes, similarly to the results of the CAMINO trial. The developments in automatic segmentation are also important to note. Image segmentation algorithms identify and delineate specific anatomical structures, which can assist in image diagnostics or 3D reconstruction. The algorithms used to deliver this are long established, however, they are limited by imaging artifacts, such as noise and partial volume defects, and are not generalizable to different anatomical areas [29]. AI methods for image segmentation, such as convolutional neural networks or generative adversarial networks, can learn complex image features, increasing the segmentation accuracy and the generalizability of models [30]. The automation of this process using AI will have significant resource and cost savings for healthcare systems. With these limitations unlocked by AI, it is likely that imaging fusion will feature in routine hepato-biliary surgery planning.

**Figure 3:**
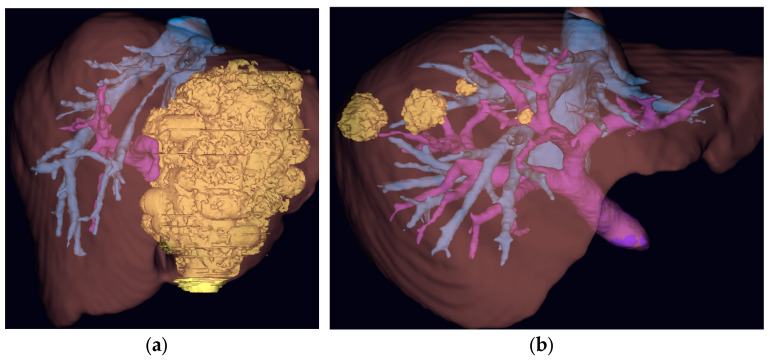
Images from HoloCare© Studio for liver surgery planning, 3D reconstructions of CRLMs using portal venous phase CT images (blue: hepatic veins; purple: portal veins; yellow: CRLM). (**a**) Single large CRLM threatening FLR; (**b**) multiple CRLMs in close proximity to hepatic and portal veins.

The utility of 3D reconstructions has been previously described in the literature for hepato-biliary surgery. Fang et al. used 3D models to plan resections for centrally located HCC in a retrospective cohort study [31]. For the 3D model intervention group (*n* = 60), a significant decrease was observed in operative time (294.5 ± 61.9 min vs. 324.3 ± 83.1 min; *p* = 0.028), rate of hepatic inflow occlusion (51.7% vs. 71.4%; *p* = 0.029) and major complication rate (3.3% vs. 14.3%; *p* = 0.048) compared to the control group. Although this is early evidence, it provides a signal towards improved outcomes because of enhanced pre-operative planning. With medical software for 3D reconstruction of hepato-biliary anatomy becoming more readily available, it is likely that further evidence of benefit will be generated in the near future (Figure 3). Another possible advantage involves the uptake of minimally invasive surgery. It is possible that enhanced visualization using 3D reconstructions facilitates an increase in laparoscopic or robotic approaches to hepato-biliary surgery, which are known to positively influence patient outcomes. In conclusion, GenAI has the potential to revolutionize patient selection, combining multiple data inputs, including established clinical parameters and novel data points, such as wearable device recordings, to generate accurate risk prediction outputs for clinicians. These outputs may include MDT recommendations, providing on-demand decision aids for healthcare teams. AI is also set to enhance pre-operative imaging, enabling automatic fusion of multiple imaging modalities, to create a single- and fused-image output that aids anatomical understanding. These outputs can be displayed in 3D models, which are already showing an early signal of benefit for pre-operative planning.

## 4. Extended Reality and Navigation Systems

### 4.1. Immersive Technologies

**Figure 4:**
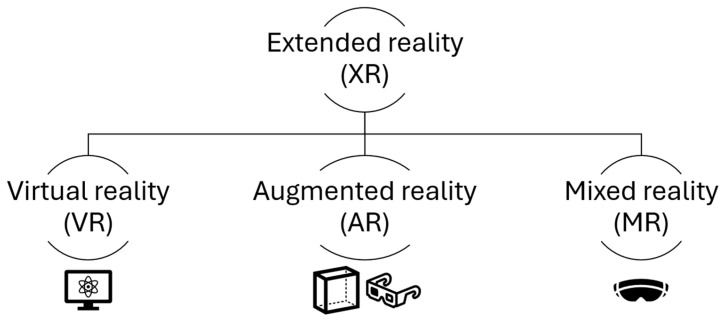
Graphical illustration of extended reality technology.

A new technology gaining large interest in healthcare is XR. XR is an umbrella term encompassing all forms of new devices that alter the human-computer interaction [32] (Figure 4). This includes virtual reality (VR), where the user sees digitally rendered images without the physical world, augmented reality (AR), involving the physical world augmented by digital information and mixed reality (MR), which uses a mixture of methods to blend the physical and digital world for the user. A potential use involves the display of 3D holograms for surgical planning. Hepato-biliary surgeons require ample training to be able to visualize complex anatomy using a 2D scan to formulate a mental 3D image. The 3D visualization technology eliminates this step and with XR enables the surgeon to interact with individual patient anatomy in an immersive environment. This could enhance the pre-operative planning through a better understanding of patient anatomy. The next stage involves bringing XR to the operating theatre and utilizing the imaging abilities for surgical navigation.

### 4.2. Surgical Navigation Systems

3D model display using XR can now be used in the operating theatre. Holograms can be displayed in a variety of ways tailored to the operative approach, using direct overlay onto the patient in open surgery, virtual display on a laparoscopic monitor or using mixed reality that is fully integrated within a surgical robotic platform. This enables the hepato-biliary surgeon to visualize specific anatomy in the operating theatre, anticipating key steps such as vascular ligation or tumor dissection. We hypothesize that this in turn leads to more efficient operating, as the surgeon progresses with greater confidence, reducing operative times. Ultimately, XR navigation systems could decrease complication rates and improve cancer resection margins through improved operative performance. The potential for improved patient outcomes is substantial. Early XR navigations systems have already been utilized and documented in the literature. Table 1 summarizes examples of studies which have evaluated XR surgical navigation systems for hepato-biliary surgery.

**Table 1:** Clinical studies using XR technology for surgical navigation in hepato-biliary surgery.

Ref.	*n*	Intervention/Surgery Performed	Results	Significant Findings
[33]				
	85	Augmented reality navigation system/laparoscopic anatomical hepatectomy for primary liver cancer	Length of stay: Intervention = 7 days Control = 10 days *p* = 0.003	Decreased length of stay and estimated blood loss in the augmented reality group
			Estimated blood loss: Intervention = 200 mL Control = 300 mL *p* = 0.002	
[34]	45	Mixed reality navigation combined with intra-operative ultrasound/laparoscopic anatomical hepatectomy for primary liver cancer	Estimated blood loss: Intervention = 103 mL Control = 259 mL *p* < 0.001 Complication rates: Intervention = 1 Control = 7 *p* = 0.021	Decreased estimated blood loss, complication rates and operative time in the mixed reality group
			Operative time: Intervention = 135 min Control = 199 min *p* < 0.001	
[35]	7	Augmented reality navigation for pancreaticoduodenectomy	Estimated blood loss: Intervention = 901 mL Control = 825 mL *p* > 0.05	No significant differences
			Operative time: Intervention = 412 min Control = 425 min *p* > 0.05	
[36]	27	Augmented reality navigation for laparoscopic cholecystectomy	Estimated blood loss: Intervention = 0 mL Control = 0 mL *p* > 0.05	No significant differences
			Operative time: Intervention = 74 min Control = 58 min *p* > 0.05	

XR navigation systems have been used for a variety of hepato-biliary procedures, including liver resection, pancreaticoduodenectomy and laparoscopic cholecystectomy (Table 1). The current evidence is derived from early evaluation studies, as expected for an evolving technology at this stage. However, a signal towards improved patient outcomes for hepatectomy has been shown in some studies. The potential applications of XR show promise for hepato-biliary surgery. For example, imaging in the form of US or magnetic resonance cholangiopancreatography (MRCP) could be used to develop mixed reality holograms for laparoscopic cholecystectomy. Using AI-powered segmentation and image overlay in a laparoscopic platform, the surgeon would be able to visualize biliary anatomy in real-time, without additional preparation. This would be a useful surgical tool without the drawbacks of current visualization systems, for example ICG which requires intravenous administration. The ability to visualize biliary anatomy may decrease the risk of BDI, and therefore improve patient outcomes. The developments in XR technology now enable the hepato-biliary to interact with imaging in an immersive environment, providing a better appreciation of individual patient anatomy. XR can also be used for navigation in the operating theatre, which is already showing improvements in liver resection outcomes. The described technologies could revolutionize pre-operative planning and surgical performance for patients with hepato-biliary conditions.

## 5. Surgical Education, Training and Patient Involvement

**Figure 5:**
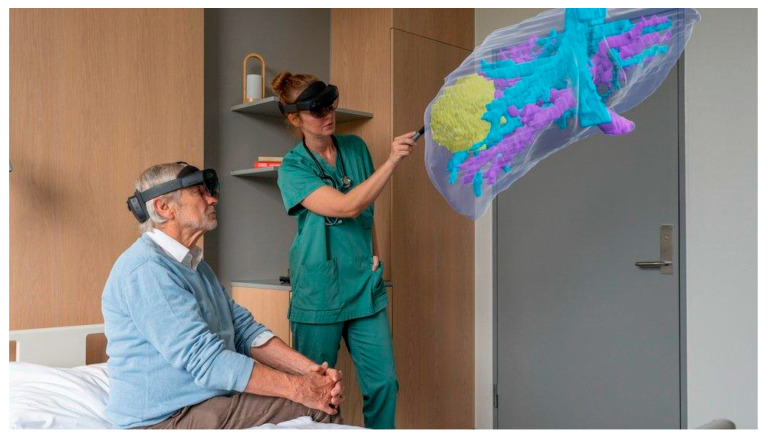
Picture illustrating patient consultation using a 3D hologram. Image from HoloCare©.

Beyond immediate clinical utility, AI and XR offer significant potential in training the next generation of surgeons. Pre-operative planning tools that use 3D visualization or AI-guided scenarios can accelerate learning curves for junior team members and improve anatomical understanding for trainees [37,38,39]. XR environments can simulate rare or complex cases, offering safe and repeatable exposure outside the operating theatre. Moreover, AI algorithms can provide objective feedback on performance, enabling data-driven progression assessments [40]. These innovations may be particularly impactful in lower-volume centers where hands-on training opportunities are limited. Furthermore, this technology may enhance patient engagement, 3D reconstructions and holographic models can be used during consultations to visually explain surgical procedures and associated risks (Figure 5). This could improve patient understanding, reduce anxiety, and foster shared decision-making [41]. Furthermore, AI tools capable of synthesizing individual risk profiles may enable more personalized discussions around expected outcomes, helping align treatment strategies with patient preferences and values. The applications of AI and XR are therefore not limited to pre-operative planning, and could be used to enhance surgical training, through simulated scenarios with objective data-driven assessments of performance. Another potential application involves patient consent, improving patient understanding through interactive 3D reconstructions of anatomy and surgical procedures.

## 6. Current Challenges and Limitations

To bring these technological advances into routine hepato-biliary practice, several current limitations must be addressed. Data is key to training accurate AI algorithms which can process complex data. Although public databases of open-source images exist (The Cancer Imaging Archive, USA), they are limited in scope, preventing model generalizability [42]. There is a need for high quality, robust and ethically and data protection- compliant databases with image data and metadata for AI training. This should be achieved through effective collaboration between different healthcare institutions, and it is already a priority for European Union (EU)-funded projects [43]. Creation of such databases will help to create accurate and effective AI models for pre-operative planning in hepato-biliary surgery. In addition to technical considerations, ethical and legal issues present critical barriers to widespread adoption. Robust data governance frameworks must ensure patient privacy, informed consent, and responsible AI usage. For instance, under the General Data Protection Regulation (GDPR) in the EU, any use of clinical data must maintain strict anonymization and transparency in data processing. Moreover, AI models trained on historical datasets must be scrutinized for potential biases that could perpetuate health inequities. The development of explainable AI algorithms is therefore essential to support clinician trust and accountability in clinical decision-making. Another area to address is standardization of research. As the fields of AI and XR involve rapidly evolving technology, safety of novel interventions must be ensured through effective research reporting. Loftus et al. reviewed 36 studies exploring AI decision support for surgery and found that a minority of research reported external validation (13.8%), real-time validation (5.6%) and clinical implementation frameworks (36.1%). Innovation in surgery should be reported in line with established frameworks, such as the Idea, Development, Exploration, Assessment and Long-term follow-up (IDEAL) framework, to develop robust evidence of clinical benefit for patients [44]. Accordingly, various frameworks for reporting AI in research, such as CONSORT-AI or DECIDE-AI, have been developed and their use should be employed more widely for surgical decision-making research [45]. Currently, there are no available frameworks for XR studies in surgery and this should be developed to facilitate safe and effective research. Another limitation relates to the early development stage of these technologies. The research described in this review features mostly interventions confined to a single clinical condition and usually involving a specific clinical intervention (such as decision-aid or mixed reality display of images). To facilitate wider adoption of AI and XR, technologies must be developed to address inter-disciplinary clinical problems. For example, a platform for MDT decision-making should involve different cancers and oncological subspecialities in one system. In the case of XR, interventions should be developed to work across different surgical laparoscopic and robotic platforms. Tools enabling decision-assistance for multiple clinical problems in different healthcare environments are more likely to be implemented, leading to wider adoption.

The limitations outlined can be addressed with further technological developments and research. As with any new technology, cost is a concern for healthcare systems when considering adoption into clinical practice. Economic considerations are pivotal in determining the real-world impact of AI and XR innovations. Upfront costs for infrastructure, software and training can be substantial. However, these may be offset by long-term gains, such as reduced complication rates, shorter operative times, lower readmission rates and substantial gain of clinical experience. Cost-effectiveness analyses comparing traditional planning with AI-assisted or XR-integrated pathways will be important to support policy decisions, especially in resource-constrained settings. Additionally, equitable access must be considered to prevent the emergence of a technological divide across surgical institutions. An additional benefit of AI and XR to consider in the cost-effectiveness analysis is in surgical training. For pre-operative planning, many years of clinical experience are required to process patient data and images to formulate appropriate surgical plans. We hypothesize that AI and XR assistance will be of most value to the early-career hepato-biliary surgeon. There is a well-documented association between the experience of a surgeon and patient outcomes when undergoing hepato-biliary surgery [46,47]. The ability to generate decision aids and operative plans through the use of AI and XR could accelerate the learning of an early-career hepato-biliary surgeon. Although the cost–benefit may not be seen in experienced centers, the benefits for surgeons building their clinical experience should be investigated in future research and cost analysis.

## 7. Conclusions

Pre-operative planning for hepato-biliary surgery is essential in ensuring appropriate decision-making for patients and the best possible outcomes. Advances in AI and XR technologies provide new tools for the hepato-biliary surgeon. AI could soon be used to provide on-demand and accurate risk prediction and treatment plans for patients. Traditional 2D images from different modalities could be transformed using AI into 3D holograms involving fused images from CT, MRI and US scans. These can now be displayed in an immersive environment using XR technology, enabling the clinician to interact with the anatomy, make surgical plans and navigate in the operating theatre. Both technologies could be used to simulate rare and challenging scenarios for surgeons in training, accelerating learning curves. Limitations include data availability, ethical considerations, research reporting and lack of inter-disciplinary AI/XR platforms. These must be addressed to improve the development and adoption of these new technologies. The future of pre-operative hepato-biliary planning will transform patient care, empowering clinicians and patients with more data to facilitate the best possible outcomes.

## Abbreviations

The following abbreviations are used in this manuscript:

AI	Artificial Intelligence
CRLM	Colorectal Liver Metastasis
CALI	Chemotherapy-Associated Liver Injury
GenAI	Generative Artificial Intelligence
HCC	Hepatocellular Carcinoma
XR	Extended Reality
PHLF	Post-hepatectomy liver failure
HPB	Hepato-pancreato-biliary
FLR	Future Liver Remnant
AUC	Area Under Curve
CT	Computed Tomography
MRI	Magnetic Resonance Imaging
MRCP	Magnetic Resonance Cholangiopancreatography
US	Ultrasound
